# 2,3-Bis(prop-2-yn­yloxy)naphthalene

**DOI:** 10.1107/S1600536808037549

**Published:** 2008-11-20

**Authors:** Guo-Zhong Yang, Fu-An Li

**Affiliations:** a College of Civil and Architectural Engineering, Henan University, Kaifeng 475001, Henan, People’s Republic of China; b College of Chemistry and Chemical Engineering, Pingdingshan University, Pingdingshan 467000, Henan, People’s Republic of China

## Abstract

In the crystal structure of the title compound, C_16_H_12_O_2_, no classical hydrogen bonds or aromatic π–π stacking inter­actions were observed. The mol­ecules are linked into a three-dimensional framework by a combination of C—H⋯O and C—H⋯π(arene) hydrogen bonds.

## Related literature

For related structures, see: Zhang *et al.* (2008 ▶); Ghosh *et al.* (2007 ▶); Wang & Kong (2007 ▶). For the synthesis, see: Burchell *et al.* (2006 ▶). For bond-length data, see: Allen *et al.* (1987 ▶). For π–π stacking inter­actions, see: Steed & Atwood (2000 ▶).
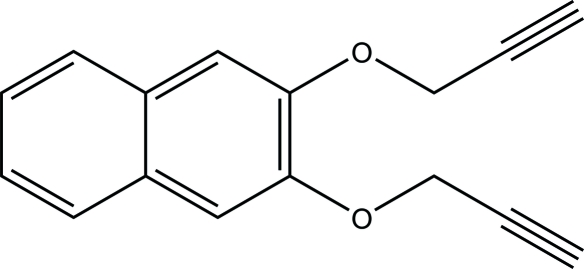


## Experimental

### 

#### Crystal data

C_16_H_12_O_2_
*M* *_r_* = 236.26
Orthorhombic, 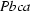

*a* = 8.2921 (12) Å
*b* = 9.0457 (14) Å
*c* = 33.070 (5) Å
*V* = 2480.5 (6) Å^3^
*Z* = 8
Mo *K*α radiation
μ = 0.08 mm^−1^
*T* = 298 (2) K
0.18 × 0.16 × 0.15 mm

#### Data collection

Bruker SMART APEXII CCD area-detector diffractometer
Absorption correction: multi-scan (*SADABS*; Bruker, 2005 ▶) *T*_min_ = 0.984, *T*_max_ = 0.989
12468 measured reflections
2182 independent reflections
1782 reflections with *I* > 2σ(*I*)
*R*_int_ = 0.033

#### Refinement

*R*[*F* ^2^ > 2σ(*F* ^2^)] = 0.039
*wR*(*F* ^2^) = 0.101
*S* = 1.04
2182 reflections
163 parameters
H-atom parameters constrained
Δρ_max_ = 0.17 e Å^−3^
Δρ_min_ = −0.19 e Å^−3^

### 

Data collection: *APEX2* (Bruker, 2005 ▶); cell refinement: *APEX2*; data reduction: *SAINT* (Bruker, 2005 ▶); program(s) used to solve structure: *SHELXS97* (Sheldrick, 2008 ▶); program(s) used to refine structure: *SHELXL97* (Sheldrick, 2008 ▶); molecular graphics: *SHELXTL* (Sheldrick, 2008 ▶); software used to prepare material for publication: *SHELXTL*.

## Supplementary Material

Crystal structure: contains datablocks I, global. DOI: 10.1107/S1600536808037549/bv2111sup1.cif

Structure factors: contains datablocks I. DOI: 10.1107/S1600536808037549/bv2111Isup2.hkl

Additional supplementary materials:  crystallographic information; 3D view; checkCIF report

## Figures and Tables

**Table 1:** Hydrogen-bond geometry (Å, °) *Cg*1 is the centroid of the C4–C6/C11–C13 ring.

*D*—H⋯*A*	*D*—H	H⋯*A*	*D*⋯*A*	*D*—H⋯*A*
C16—H16⋯O2^i^	0.93	2.48	3.409 (2)	177
C3—H3*A*⋯*Cg*1^ii^	0.97	2.95	3.634 (2)	129

Symmetry codes: (i) 
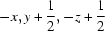
; (ii) 
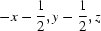
.

**Fig. 1.**
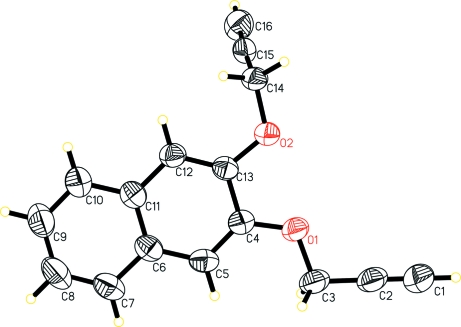
A view of the title compound, showing the atom-labeling scheme. Displacement ellipsoids are drawn at the 50% probability level and H atoms are shown as small spheres of arbritary radii.

**Fig. 2.**
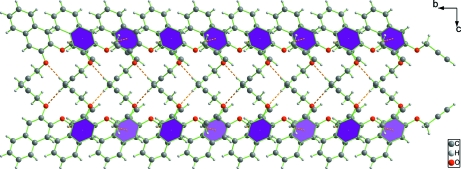
The three-dimensional supramolecular framework of the title complound formed by C—H···O and C—H···π (arene) hydrogen bonds, viewed along the a axis.

**Fig. 3.**
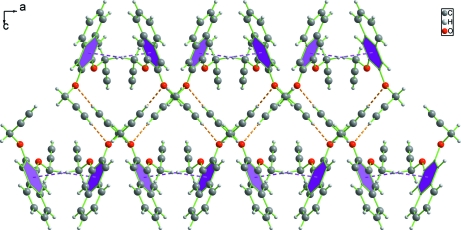
The three-dimensional supramolecular framework of the title complound formed by C—H···O and C—H···π (arene) hydrogen bonds, viewed along the b axis.

## supplementary crystallographic information

### Comment

The title compound has been characterized by *X*–ray methods (Fig. 1). The bond lengths and angles are within normal ranges (Allen *et al.* 1987). Except for an ethinyl groug [–C15≡C16–H16], all the remaining non–H atoms are almost coplanar, with a mean deviation from the least-square plane to be 0.0402 (14) Å. The angle between the ethinyl and the plane is 23.80 (9)°.

While π—π stacking interactions are often found in aromatics (Wang *et al.* 2007), in the title complex the minimal distance between ring centroids is 5.188 (1) Å indicating that there are no π—π stacking interactions present(Steed *et al.* 2000).

The molecules of the title complex are linked into a three-dimensional framework by a combination of C—H···O and C—H···π (arene) hydrogen bonds (Fig. 2, Fig. 3, Table 1). [*Cg*1 id the centroid of the C4–C6/C11–C13 ring. Symmetry codes: (i) -*x*, *y* + 1/2, -*z* + 1/2; (ii) -*x* - 1/2, *y* - 1/2, *z* + 2.]

### Experimental

The title compound was obtained unintentionally during an attempted synthesis of a network complex (Burchell *et al.*, 2006) based on Co(II) and 2,3-bis(prop-2-ynyloxy)naphthalene, involving the evaporation of a methyl alchol and acetone solution of CoCl~2~, NaN~3~ and the title molecule, at 298 K.

### Refinement

All the H atoms could be detected in the difference electron density maps. Nevertheless, they were situated into the idealized position and refined using a riding model. C—H = 0.97 Å for the methylene groups and C—H = 0.93 Å for the remaining H atoms. *U*_iso_(H) = 1.2 *U*_eq_ (carrier C) for all the H atoms.

### Crystal data

**Table:**

C_16_H_12_O_2_	*F*_000_ = 992
*M**_r_* = 236.26	*D*_x_ = 1.265 Mg m^−^^3^
Orthorhombic, *P**b**c**a*	Mo *K*α radiation λ = 0.71073 Å
Hall symbol: -P 2ac 2ab	Cell parameters from 3821 reflections
*a* = 8.2921 (12) Å	θ = 2.8–25.7º
*b* = 9.0457 (14) Å	µ = 0.08 mm^−^^1^
*c* = 33.070 (5) Å	*T* = 298 (2) K
*V* = 2480.5 (6) Å^3^	Block, colourless
*Z* = 8	0.18 × 0.16 × 0.15 mm

### Data collection

**Table:**

Bruker SMART APEXII CCD area-detector diffractometer	2182 independent reflections
Radiation source: fine-focus sealed tube	1782 reflections with *I* > 2σ(*I*)
Monochromator: graphite	*R*_int_ = 0.033
*T* = 298(2) K	θ_max_ = 25.0º
ω scans	θ_min_ = 2.5º
Absorption correction: multi-scan(SADABS; Bruker, 2005)	*h* = −9→9
*T*_min_ = 0.984, *T*_max_ = 0.989	*k* = −10→10
12468 measured reflections	*l* = −39→29

### Refinement

**Table:**

Refinement on *F*^2^	Secondary atom site location: difference Fourier map
Least-squares matrix: full	Hydrogen site location: inferred from neighbouring sites
*R*[*F*^2^ > 2σ(*F*^2^)] = 0.039	H-atom parameters constrained
*wR*(*F*^2^) = 0.101	*w* = 1/[σ^2^(*F*_o_^2^) + (0.0527*P*)^2^ + 0.338*P*] where *P* = (*F*_o_^2^ + 2*F*_c_^2^)/3
*S* = 1.04	(Δ/σ)_max_ < 0.001
2182 reflections	Δρ_max_ = 0.17 e Å^−^^3^
163 parameters	Δρ_min_ = −0.19 e Å^−^^3^
Primary atom site location: structure-invariant direct methods	Extinction correction: none

### Special details

**Table:**

Geometry. All e.s.d.'s (except the e.s.d. in the dihedral angle between two l.s. planes) are estimated using the full covariance matrix. The cell e.s.d.'s are taken into account individually in the estimation of e.s.d.'s in distances, angles and torsion angles; correlations between e.s.d.'s in cell parameters are only used when they are defined by crystal symmetry. An approximate (isotropic) treatment of cell e.s.d.'s is used for estimating e.s.d.'s involving l.s. planes.
Refinement. Refinement of *F*^2^ against ALL reflections. The weighted *R*-factor *wR* and goodness of fit *S* are based on *F*^2^, conventional *R*-factors *R* are based on *F*, with *F* set to zero for negative *F*^2^. The threshold expression of *F*^2^ > σ(*F*^2^) is used only for calculating *R*-factors(gt) *etc*. and is not relevant to the choice of reflections for refinement. *R*-factors based on *F*^2^ are statistically about twice as large as those based on *F*, and *R*- factors based on ALL data will be even larger.

### Fractional atomic coordinates and isotropic or equivalent isotropic displacement parameters (Å^2^)

**Table:**

	*x*	*y*	*z*	*U*_iso_*/*U*_eq_	
C1	−0.1360 (2)	−0.0848 (2)	0.32753 (6)	0.0752 (6)	
H1	−0.1425	−0.1672	0.3108	0.090*	
C2	−0.1277 (2)	0.01867 (18)	0.34856 (5)	0.0574 (4)	
C3	−0.1181 (2)	0.14633 (16)	0.37505 (5)	0.0554 (4)	
H3A	−0.2249	0.1865	0.3798	0.066*	
H3B	−0.0720	0.1182	0.4009	0.066*	
C4	0.00170 (15)	0.38601 (15)	0.37523 (4)	0.0400 (3)	
C5	−0.05860 (17)	0.42250 (16)	0.41224 (4)	0.0462 (4)	
H5	−0.1182	0.3532	0.4267	0.055*	
C6	−0.03174 (17)	0.56457 (16)	0.42903 (4)	0.0451 (4)	
C7	−0.0984 (2)	0.60874 (19)	0.46649 (4)	0.0586 (4)	
H7	−0.1609	0.5422	0.4811	0.070*	
C8	−0.0727 (3)	0.7468 (2)	0.48143 (5)	0.0703 (5)	
H8	−0.1182	0.7741	0.5060	0.084*	
C9	0.0218 (2)	0.8480 (2)	0.45997 (5)	0.0696 (5)	
H9	0.0401	0.9417	0.4706	0.084*	
C10	0.0875 (2)	0.80997 (18)	0.42353 (5)	0.0577 (4)	
H10	0.1500	0.8782	0.4095	0.069*	
C11	0.06147 (17)	0.66812 (16)	0.40699 (4)	0.0443 (3)	
C12	0.12274 (17)	0.62803 (15)	0.36856 (4)	0.0436 (3)	
H12	0.1830	0.6959	0.3538	0.052*	
C13	0.09452 (16)	0.49128 (15)	0.35298 (4)	0.0391 (3)	
C14	0.24207 (18)	0.53826 (15)	0.29190 (4)	0.0447 (3)	
H14A	0.3080	0.4809	0.2735	0.054*	
H14B	0.3139	0.5947	0.3092	0.054*	
C15	0.14206 (18)	0.64031 (16)	0.26860 (4)	0.0448 (4)	
C16	0.0667 (2)	0.71916 (18)	0.24743 (5)	0.0575 (4)	
H16	0.0073	0.7814	0.2307	0.069*	
O1	−0.01832 (12)	0.25415 (10)	0.35571 (3)	0.0479 (3)	
O2	0.14871 (12)	0.43928 (10)	0.31646 (3)	0.0471 (3)	

### Atomic displacement parameters (Å^2^)

**Table:**

	*U*^11^	*U*^22^	*U*^33^	*U*^12^	*U*^13^	*U*^23^
C1	0.0882 (14)	0.0598 (11)	0.0778 (12)	−0.0163 (10)	0.0254 (11)	−0.0032 (10)
C2	0.0584 (10)	0.0467 (9)	0.0672 (10)	−0.0065 (7)	0.0180 (8)	0.0088 (8)
C3	0.0586 (10)	0.0444 (8)	0.0631 (9)	−0.0032 (7)	0.0164 (8)	0.0104 (7)
C4	0.0354 (7)	0.0398 (7)	0.0448 (8)	0.0031 (6)	0.0005 (6)	0.0047 (6)
C5	0.0454 (8)	0.0499 (8)	0.0433 (8)	0.0030 (7)	0.0040 (6)	0.0117 (7)
C6	0.0448 (8)	0.0544 (9)	0.0361 (7)	0.0091 (7)	−0.0050 (6)	0.0042 (6)
C7	0.0660 (10)	0.0696 (11)	0.0403 (8)	0.0089 (8)	0.0054 (8)	0.0055 (8)
C8	0.0896 (14)	0.0803 (13)	0.0410 (8)	0.0154 (11)	0.0053 (9)	−0.0092 (8)
C9	0.0884 (13)	0.0660 (11)	0.0545 (10)	0.0014 (10)	−0.0006 (9)	−0.0156 (9)
C10	0.0643 (10)	0.0572 (9)	0.0517 (9)	−0.0036 (8)	−0.0007 (8)	−0.0081 (7)
C11	0.0418 (8)	0.0508 (8)	0.0403 (7)	0.0042 (6)	−0.0051 (6)	−0.0007 (6)
C12	0.0418 (8)	0.0453 (8)	0.0438 (8)	−0.0042 (6)	0.0031 (6)	0.0029 (6)
C13	0.0345 (7)	0.0440 (7)	0.0388 (7)	0.0025 (6)	0.0021 (6)	0.0022 (6)
C14	0.0452 (8)	0.0438 (7)	0.0453 (7)	−0.0025 (6)	0.0115 (7)	0.0018 (6)
C15	0.0497 (8)	0.0442 (8)	0.0405 (7)	−0.0073 (7)	0.0029 (7)	−0.0073 (6)
C16	0.0616 (10)	0.0558 (9)	0.0552 (9)	−0.0044 (8)	−0.0124 (8)	0.0009 (8)
O1	0.0499 (6)	0.0404 (5)	0.0535 (6)	−0.0050 (4)	0.0135 (5)	0.0027 (4)
O2	0.0535 (6)	0.0423 (5)	0.0454 (5)	−0.0060 (5)	0.0140 (5)	−0.0010 (4)

### Geometric parameters (Å, °)

**Table:**

C1—C2	1.168 (2)	C8—H8	0.9300
C1—H1	0.9300	C9—C10	1.367 (2)
C2—C3	1.452 (2)	C9—H9	0.9300
C3—O1	1.4299 (16)	C10—C11	1.412 (2)
C3—H3A	0.9700	C10—H10	0.9300
C3—H3B	0.9700	C11—C12	1.416 (2)
C4—C5	1.363 (2)	C12—C13	1.3603 (19)
C4—O1	1.3663 (16)	C12—H12	0.9300
C4—C13	1.4285 (18)	C13—O2	1.3718 (15)
C5—C6	1.418 (2)	C14—O2	1.4354 (16)
C5—H5	0.9300	C14—C15	1.461 (2)
C6—C7	1.414 (2)	C14—H14A	0.9700
C6—C11	1.416 (2)	C14—H14B	0.9700
C7—C8	1.360 (2)	C15—C16	1.179 (2)
C7—H7	0.9300	C16—H16	0.9300
C8—C9	1.399 (3)		
			
C2—C1—H1	180.0	C10—C9—H9	119.8
C1—C2—C3	179.39 (17)	C8—C9—H9	119.8
O1—C3—C2	107.72 (12)	C9—C10—C11	120.64 (16)
O1—C3—H3A	110.2	C9—C10—H10	119.7
C2—C3—H3A	110.2	C11—C10—H10	119.7
O1—C3—H3B	110.2	C10—C11—C12	121.72 (14)
C2—C3—H3B	110.2	C10—C11—C6	119.03 (13)
H3A—C3—H3B	108.5	C12—C11—C6	119.23 (13)
C5—C4—O1	126.24 (12)	C13—C12—C11	120.74 (13)
C5—C4—C13	119.93 (13)	C13—C12—H12	119.6
O1—C4—C13	113.83 (11)	C11—C12—H12	119.6
C4—C5—C6	120.91 (13)	C12—C13—O2	126.08 (12)
C4—C5—H5	119.5	C12—C13—C4	120.25 (12)
C6—C5—H5	119.5	O2—C13—C4	113.67 (11)
C7—C6—C11	118.51 (14)	O2—C14—C15	112.73 (12)
C7—C6—C5	122.53 (14)	O2—C14—H14A	109.0
C11—C6—C5	118.93 (12)	C15—C14—H14A	109.0
C8—C7—C6	121.10 (16)	O2—C14—H14B	109.0
C8—C7—H7	119.4	C15—C14—H14B	109.0
C6—C7—H7	119.4	H14A—C14—H14B	107.8
C7—C8—C9	120.29 (16)	C16—C15—C14	175.37 (15)
C7—C8—H8	119.9	C15—C16—H16	180.0
C9—C8—H8	119.9	C4—O1—C3	117.03 (11)
C10—C9—C8	120.41 (17)	C13—O2—C14	117.45 (10)
			
O1—C4—C5—C6	−178.85 (12)	C10—C11—C12—C13	−179.18 (14)
C13—C4—C5—C6	0.5 (2)	C6—C11—C12—C13	−0.7 (2)
C4—C5—C6—C7	177.07 (13)	C11—C12—C13—O2	−179.26 (12)
C4—C5—C6—C11	−1.0 (2)	C11—C12—C13—C4	0.2 (2)
C11—C6—C7—C8	−0.7 (2)	C5—C4—C13—C12	−0.1 (2)
C5—C6—C7—C8	−178.81 (15)	O1—C4—C13—C12	179.33 (12)
C6—C7—C8—C9	−0.5 (3)	C5—C4—C13—O2	179.43 (12)
C7—C8—C9—C10	0.9 (3)	O1—C4—C13—O2	−1.12 (16)
C8—C9—C10—C11	−0.2 (3)	C5—C4—O1—C3	3.1 (2)
C9—C10—C11—C12	177.43 (15)	C13—C4—O1—C3	−176.35 (12)
C9—C10—C11—C6	−1.0 (2)	C2—C3—O1—C4	177.75 (12)
C7—C6—C11—C10	1.4 (2)	C12—C13—O2—C14	−1.0 (2)
C5—C6—C11—C10	179.60 (13)	C4—C13—O2—C14	179.51 (11)
C7—C6—C11—C12	−177.05 (13)	C15—C14—O2—C13	−81.84 (15)
C5—C6—C11—C12	1.1 (2)		

### Hydrogen-bond geometry (Å, °)

**Table:**

*D*—H···*A*	*D*—H	H···*A*	*D*···*A*	*D*—H···*A*
C16—H16···O2^i^	0.93	2.48	3.409 (2)	177
C3—H3A···Cg1^ii^	0.97	2.95	3.634 (2)	129

Symmetry codes: (i) −*x*, *y*+1/2, −*z*+1/2; (ii) −*x*−1/2, *y*−1/2, *z*.
